# Cost-effectiveness analysis of mindfulness-based cognitive therapy in patients with anxiety disorders in secondary mental health care settings alongside a randomized controlled trial

**DOI:** 10.3389/fpsyt.2024.1391786

**Published:** 2024-10-25

**Authors:** Mitsuhiro Sado, Akihiro Koreki, Akira Ninomiya, Chika Kurata, Sunre Park, Daisuke Fujisawa, Teppei Kosugi, Maki Nagaoka, Atsuo Nakagawa, Masaru Mimura

**Affiliations:** ^1^ Keio University Health Center, Tokyo, Japan; ^2^ Department of Neuropsychiatry, Keio University School of Medicine, Tokyo, Japan; ^3^ Keio University Mindfulness and Stress Research Center, Tokyo, Japan; ^4^ Department of Psychiatry, National Hospital Organization Shimofusa Psychiatric Medical Center, Chiba, Japan; ^5^ Faculty of Nursing and Medical Care, Keio University, Tokyo, Japan; ^6^ Division of Patient Safety, Keio University Hospital, Tokyo, Japan; ^7^ Department of Neuropsychiatry, St. Marianna University School of Medicine, Kawasaki, Japan; ^8^ Department of Preventive Medicine and Public Health, Keio University School of Medicine, Tokyo, Japan

**Keywords:** mindfulness, cost-effectiveness, mindfulness-based cognitive therapy, anxiety disorders, randomized controlled trial

## Abstract

**Introduction:**

Anxiety disorder is one of the most prevalent mental disorders. Mindfulness-based cognitive therapy (MBCT) is effective for treating anxiety disorders. However, no studies have investigated the cost-effectiveness of MBCT for anxiety disorders. We aimed to conduct a cost-effectiveness analysis alongside a randomized controlled trial (RCT) to clarify the cost-effectiveness of MBCT for anxiety disorders.

**Methods:**

A cost-effectiveness analysis alongside an RCT was conducted for 8 weeks in 40 patients with anxiety disorders at a university hospital. Patients (1) aged 20–75 years; (2) who were diagnosed with panic disorder/agoraphobia or social anxiety disorder based on the Diagnostic and Statistical Manual of Mental Disorders, Fourth Edition, criteria; and (3) who provided written consent were analyzed. The participants were allocated randomly (1:1 ratio) to the augmented MBCT group (i.e., MBCT plus treatment as usual [TAU]) or TAU (waitlist control) group. The cost-effectiveness was assessed using the incremental cost-effectiveness ratio (ICER), which is the ratio of the incremental costs divided by the incremental state-trait anxiety inventory- state (STAI-S), state-trait anxiety inventory- trait (STAI-T), and quality-adjusted life years (QALYs). The QALYs were estimated using The Japanese version of EuroQoL five-dimensional 3-level questionnaire. The unit cost data were derived from the government-regulated fees. This study was conducted from a public healthcare insurance perspective. No discount rates were considered.

**Results:**

A total of 38 participants with complete data were included in the analysis. The MBCT was JPY 13,885 more than the cost of TAU and was associated with a STAI-S, STAI-T, and QALY increase of 10.13, 12.00, 0.009 respectively. The ICER were JPY 1,371 (USD13) per STAI-S, JPY 1,157 (USD 11) per STAI-T, and JPY 1,566,357 (USD 14,940) per QALY respectively. MBCT had an 77.5% probability of being cost-effective at a willingness to pay threshold in Japan (JPY 5,000,000 per QALY). The results of the four one-way sensitivity analyses supported the robustness of the base-case analysis findings.

**Discussion:**

Augmented MBCT for anxiety disorders is cost-effective compared with TAU post-treatment from a public healthcare insurance perspective. Future studies should include long-term observations, and analysis from a societal perspective.

## 1 Introduction

Anxiety disorder is one of the most prevalent mental disorders. The 12-month prevalence rates in 2021 were 7.68% in the United States, 7.36% in Europe, and 3.34% in Japan (1). It was more prevalent than depression (6.06%, 5.70%, and 3.15%, respectively), and had a considerable impact on the Global Burden of Disease (2). This burden can be expressed in terms of the monetary costs associated with a given disease. Previous studies revealed that anxiety disorders can impose a substantial economic burden on the afflicted and society as a whole [42.3 billion United States dollars [USD] in 1990 in the US, (3), 8.9 billion British pounds [GBP] in 2007 in England (4), and 2.4 trillion Japanese yen [JPY] in 2008 in Japan (5)].

Clinical guidelines commonly recommend pharmacotherapy and cognitive behavioral therapy (CBT) as the first-line treatments for anxiety disorders (6–8). However, in Japan, the shortage of CBT specialists makes it difficult to perform individual CBT for all suitable patients although most patients prefer this treatment, if available (9). As a result, individual CBT effectively becomes a second-line treatment option. Even so, the number of patients who can receive individual CBT as a second-line treatment is still limited. Therefore, to improve access to psychological treatment, more efficient methods (e.g., group psychotherapy) should be considered before proceeding with more resource-intensive interventions (e.g., individual CBT) as part of a stepped-care approach.

Mindfulness-based cognitive therapy (MBCT) is a promising group psychotherapy for anxiety disorders, which was originally designed to prevent the recurrence of depression (10). Numerous studies have demonstrated its clinical effectiveness in various conditions (e.g., depression, chronic pain, and cancer-associated distress) (11–22), and anxiety is not an exception. A recent meta-analytical review revealed that mindfulness-based intervention (MBI) is effective for anxiety disorders (23). This finding was also observed in a Japanese study; Ninomiya et al. reported that MBCT is effective in patients with anxiety disorders in secondary mental healthcare settings (24).

To expand access to such evidence-based psychological interventions (25), studies investigating the cost-effectiveness of MBCT for anxiety disorders are required. According to recent systematic reviews (26, 27), only one study performed a cost-effectiveness analysis of the MBI associated with anxiety (28). However, as this trial was performed in a patient population with various diagnoses (i.e., depression, anxiety, stress, and adjustment disorders), assessment for anxiety disorders was not possible. To the best of our knowledge, no study has examined the cost-effectiveness of MBIs for anxiety disorders. Therefore, we aimed to conduct a cost-effectiveness analysis alongside a randomized controlled trial (RCT) to clarify the cost-effectiveness of MBCT for anxiety disorders. Among various types of anxiety disorders, we decided to target individuals with panic disorder and social anxiety disorder in this study. Considering that MBCT is designed for the remission state of depression, these disorders, characterized by intermittent symptoms, are appropriate for our focus.

## 2 Methods

We conducted a cost-effectiveness analysis alongside an RCT. This RCT assessed the effectiveness of MBCT in patients with anxiety disorders in secondary mental healthcare settings. The details of the effectiveness study are described separately (24). This study was approved by the Ethics Review Committee of the Keio University School of Medicine (ID: 20140100) and was conducted in accordance with the Consolidated Standards of Reporting Trials statement (29, 30) and Consolidated Health Economic Evaluation Reporting Standards guidelines (31).

### 2.1 Setting and location

Participants from the outpatient division of the Department of Neuropsychiatry at the Keio University School of Medicine in Tokyo were recruited for the study between September 2014 and May 2015.

### 2.2 Design

The clinical trial was performed as a pragmatic 8-week RCT, comparing augmented MBCT and treatment as usual (TAU) for anxiety disorders with a nested cost-effectiveness analysis.

### 2.3 Participants

Patients who (1) were aged between 20 and 75 years; (2) met the diagnostic criteria for panic disorder/agoraphobia or social anxiety disorder specified in the Diagnostic and Statistical Manual of Mental Disorders, Fourth Edition; and (3) were able to provide written consent were included in the study. For diagnostic assessment, we used the Japanese version of the Structured Clinical Interview for DSM-IV Axis I Disorders. Meanwhile, patients with (1) a history of substance abuse or dependence, (2) organic brain damage, (3) cognitive dysfunction, (4) current or past episodes of psychosis (including bipolar disorder), (5) antisocial personality disorder, (6) severe physical problems, and (7) suicidal behaviors were excluded. Those who had been previously offered MBIs or who were not expected to attend more than four sessions (e.g. planned relocation) were also excluded. If a participant decided to withdraw from the program and the assessments, he or she was defined as a dropout.

### 2.4 Randomization and masking

The participants were allocated randomly (1:1 ratio) to either the augmented MBCT group (i.e., MBCT plus TAU) or the waitlist control group (i.e., receiving TAU during the waiting period, and received MBCT after the waiting period completed). A computer-generated random number stratified based on the baseline state-trait anxiety inventory (STAI) state anxiety subscale score (<40 or ≥40) and the anxiety disorder diagnosis (panic disorder/agoraphobia or social anxiety disorder) was used for random allocation to blind the allocation status. The Keio Center for Clinical Research Project Management Office, Tokyo, Japan, performed this process independently of the research team. Owing to the nature of the intervention, we could not conceal the participants or the MBCT therapists’ allocation status.

### 2.5 Intervention

#### 2.5.1 MBCT

Participants allocated to the augmented MBCT arm underwent 2-hour MBCT sessions in group-based format (same as the original MBCT) for 8 weeks (eight sessions in total). Each group comprised 10 participants. The MBCT program for depression developed by Segal et al. was applied in this study (10), which consisted of mindfulness practices (e.g., raisin exercise, body scan, sitting meditation, mindful walking, and 3-minute breathing space) and cognitive approaches. Due to the high symptom overlap between depression and anxiety disorders, minimal modifications were made to the original program. The difference from the original program was that we skipped the 1-day silent retreat between weeks 6 and 7 and replaced the lecture related to depression with one relevant to anxiety. The detailed contents of the program can be found in the original report (24).

The participants were requested to practice mindfulness meditation daily and report their records weekly to the research team during the intervention period. The first author conducted the sessions as the primary therapist. He is a qualified mindfulness-based stress reduction (MBSR) teacher at the University of Massachusetts Medical School and has completed all modules of the MBCT teachers’ path at the Oxford Mindfulness Foundation. The third author joined the sessions as a co-therapist.

#### 2.5.2 Treatment as usual

The TAU program mainly included pharmacotherapy and a brief consultation with a specialized psychiatrist. We did not apply any specific restrictions to drug choice, drug doses, or frequency of psychiatric visits. Specific forms of individual or group psychotherapy (e.g., cognitive behavior therapy, Morita therapy, and interpersonal therapy) were not allowed during the study period. The treatments included in the TAU program differ depending on the country and context. In Japan, a specific form of psychotherapy is only offered in 0.27% of all psychiatrist visits in usual care settings (32). Therefore, the definition of TAU was considered reasonable.

### 2.6 Data collection

We assessed the clinical conditions at three time points: baseline, during the intervention (4 weeks), and at the end of the intervention (8 weeks).

### 2.7 Outcome measures

The primary outcome in the clinical trial was the mean difference in the change in the STAI scores between the groups from baseline to the end of the intervention (8 weeks); this was estimated with a self-report approach. The STAI is a commonly used measure of state and trait anxiety. It can be used in clinical settings to diagnose anxiety and to distinguish it from depressive syndromes. It has 20 items for assessing trait anxiety and 20 items for assessing state anxiety. Higher score indicates higher anxiety status (33). The following events were considered serious adverse consequences: death, life-threatening events, events leading to severe disability or functional impairment, and hospitalization. Adverse events were monitored in each session, and the participants were asked to report them to the researchers.

### 2.8 Health service use

The health service use data were collected from the clinical reports of patients at each observational time point (i.e., baseline, 4 weeks, and 8 weeks). The participants were requested to report the number/amount of each healthcare service related to the management of psychiatric diseases since the previous assessment. The healthcare services included in the analysis were psychiatrist visits, MBCT cost, and prescribed medications.

The healthcare service use data were converted into cost data by multiplying by the unit cost of each service. The unit cost derived from the data of 2023/2024. The costs were presented in both JPY and USD. The purchasing power parity ratios in 2023 (i.e., one USD) were equal to 104.84 JPY (34). The methods used in estimating each unit cost are as follows.

#### 2.8.1 Psychiatric visit cost

The cost of a psychiatric visit includes general consultant fees, psychiatric consultation fees, and prescription fees (**Table 1**). Each unit fee was defined based on a list of government-regulated fees (35).

**Table 1:** Unit cost of each healthcare service.

	Unit cost (JPY)	cost year	Source
General consultant fee (per visit)	750	2024	MHLW of Japan (35)
Psychiatric management fee (per visit)	3,150	2024	MHLW of Japan (35)
Prescription fee (per prescription)	1,050	2024	MHLW of Japan (35)
MBCT (per session per patient)	2,700	2024	MHLW of Japan (35)
MBCT (per session) (in sensitivity analysis)	32,529	2023	MHLW of Japan (41)
Mean weighted daily cost per DDD of each drug category*			
antidepressants	67.1	2024	Nakagawa et al. (38), MHLW of Japan (35), WHO (36)
anxiolytics/hypnotics	12.5	2024	Nakagawa et al. (38), MHLW of Japan (35), WHO (36)
antipsychotics	159.5	2024	Nakagawa et al. (37), MHLW of Japan (35), WHO (36)
mood stabilizer	113.0	2024	Nakagawa et al. (37), MHLW of Japan (35), WHO (36)

DDD, defined daily dose.

MHLW, Ministry of Health, Labour and Welfare.

JPY, Japanese yen.

MBCT, mindfulness-based cognitive therapy.

*The method used to calculate the mean weighted cost per DDD per day of drug category is provided in **Supplementary Table 1**.

#### 2.8.2 MBCT costs

The unit cost of MBCT was estimated to be JPY 2,700 per session per patient, which is the government-determined reimbursement fee for group psychotherapy (**Table 1**) (35).

#### 2.8.3 Medication costs

The medication costs consisted of the dispensing and medication costs. The psychotropic drugs were divided into four groups: antidepressants, anxiolytics/hypnotics, antipsychotics, and mood stabilizers. Because the prices of medications in the same category (e.g., paroxetine and sertraline in the antidepressant category) differ, the medication costs are unequal when each patient is prescribed equivalent doses of different drugs in the same category (e.g., paroxetine 40 mg and sertraline 100 mg). To adjust for this difference, the daily dose of each drug was converted into a fraction of the defined daily dose (DDD) based on the World Health Organization-assumed average maintenance daily dose estimated from the dosage recommendations for each drug (36). By multiplying this fraction by the weighted unit cost, the cost of each medicine was estimated using the following formula:


Cmed−n−d=Dn×DDDn×Cmed−c

where *Cmed-n-d, Dn, DDDn*, and *Cmed-c* represent the cost of medicine *n* at dose *d*, the dose of the prescribed medicine *n*, the DDD of medicine *n*, and the weighted cost of category *C* to which the medicine n is attributed, respectively.

We estimated the mean weighted daily medication cost per DDD for each drug category based on the findings of previous studies and government reports (35–38). The results and methods used to estimate them are provided in **Table 1** and **Supplementary Tables 1A–E**.

The total medication costs were estimated by determining the area of the two trapezoids squared by the medication costs per day and the duration of 8 weeks.

### 2.9 Economic evaluation

#### 2.9.1 Cost-effectiveness analysis

In the economic evaluation, we set the primary effectiveness outcome as STAI-S, and STAI-T. We compared the cost-effectiveness of the augmented MBCT and TAU based on the incremental cost-effectiveness ratios (ICERs), calculated as the ratio of the incremental costs divided by the decrease in STAI-S, and STAI-T score. We estimated ICER per quality adjusted life years (QALYs) as well. The QALYs were estimated as the area under the curve of the health-related utilities using the EuroQoL five-dimensional 3 level (EQ-5D-3L) questionnaire (at baseline, 4 weeks, and 8 weeks post-randomization) (39). We used the value set by the Japanese version of the EQ-5D-3L questionnaire (40) to assess health-related utilities. The observational period was set at 8 weeks post-intervention. The analysis was based on a public healthcare insurance perspective. Because the study period was short (8 weeks), the discount rate was not considered.

#### 2.9.2 Statistical analyses

The t-test for continuous variables were used for testing the treatment engagement. The significance level used in the test was 5% two-sided. Two-sided 95% confidence intervals were used when calculating confidence intervals. The estimated sample size comprised 20 participants for each arm, with one-sided significance level of 5% and statistical power of 80%, allowing for 20% attrition.

Similar to other typical cost-effectiveness analyses alongside clinical trials, the cost-effectiveness was assessed using complete samples because the QALYs required health-related utility scores at all observational points. The ICER was used to compare the augmented MBCT’s cost-effectiveness (MBCT plus TAU) with that of TAU alone. The ICER was estimated as the result of 1,000 resamplings from the original samples using the non-parametric bootstrap method. Uncertainty was assessed by drawing probabilistic acceptability curves for clinical and political decision-making. The acceptability curves represent the probability that the enhanced augmented MBCT is more cost-effective than TAU alone over a range of hypothetical values placed on the incremental outcome (willingness to pay [WTP] by the decision-makers of the healthcare system). The net monetary benefit approach was used to draw the cost-effectiveness acceptability curves. The net monetary benefit is defined as follows:

Where λ is the WTP for an incremental unit of improvement in the outcome measures, and ΔE and ΔC represent the difference in effectiveness and cost, respectively, between the groups.

#### 2.9.3 Sensitivity analysis

In order to assess the robustness of the results related to cost-effectiveness, three and four one-way sensitivity analyses for ICER per STAI, and QALY were conducted respectively. In the first analysis, the unit cost of MBCT was set as JPY 32,529 per session regardless of the number of participants. This figure was calculated by multiplying the average hourly wage of physicians in Japan in 2023 (JPY 6,506) (41) by 2 physicians and 2.5 hours (i.e. including 30 minutes for preparation and cleanup and 2 hours for the session). This analysis was aimed at determining the actual human capital cost rather than the revenue from the healthcare insurance: in Japan, the reimbursement is determined according to the “fee for service per patient” schema. In the second analysis, all samples were analyzed including those with missing data. The last observation carry forward method (LOCF) was used for the imputation of missing data. The third analysis was performed using all samples with different unit costs of MBCT (JPY 32,529 per session regardless of the number of participants). The fourth analysis focused exclusively on ICER per QALY to assess the impact of different methodologies for estimating QALYs. Rather than using the base case analysis method, which relies on the absolute area under the curve of health-related utilities, we opted for the incremental area from the baseline to account for baseline differences in the EQ-5D score. Statistical analyses were performed using R (4.0.2) (42).

## 3 Results

### 3.1 Participants

**Figure 1:**
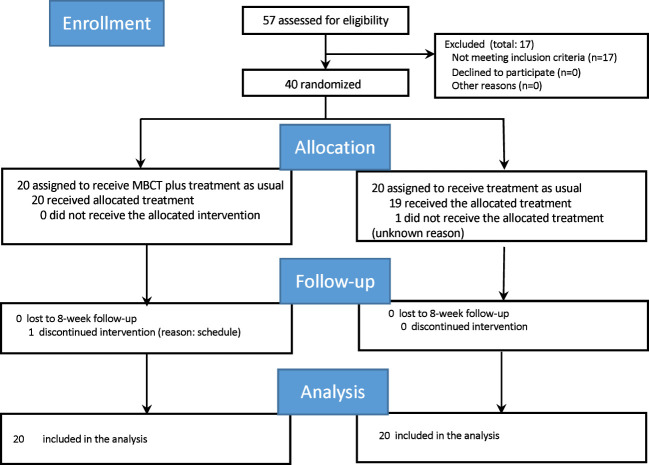
Consolidated Standards of Reporting Trials (CONSORT) diagram of participants flow through the study. MBCT, mindfulness-based cognitive therapy.

The final intervention was conducted in July 2015. The process of participant selection from screening to post-intervention is shown in **Figure 1**. Of the 57 candidate participants, 17 who did not meet the inclusion criteria were excluded. Hence, only 40 participants were randomly allocated to either the MBCT plus TAU group or TAU alone (waitlist group) group. The attrition rates were the same in both groups (one in each group).

As in the original article, the average duration of disorders from onset (mean [standard deviation (SD)]) was 151.2 [123.3] months, and the average treatment duration (mean [SD]) was 110.7 [118.7] months. Approximately 95% of all participants were prescribed at least one psychotropics (2.9 psychotropics on average), while 67.5% were prescribed at least one antidepressant at baseline. No significant differences were observed in any of the variables between the groups, including age, sex, or diagnosis. No significant differences were also observed in the clinical measures and average duration of anxiety disorders from the onset or treatment initiation.

### 3.2 Treatment engagement and health service use

One participant in each arm (two in total) dropped out before the study was completed, one in the augmented MBCT group withdrew from the study during the intervention period (after receiving the first session), and one in the control group discontinued after the baseline assessment. The average number of MBCT sessions attended (mean [SD]) was 6.8 [2.2].

Although the difference was not significant (p=0.262), the number of psychiatrist visits during the study period was slightly higher in the control group (mean [SD]: 3.5 [2.0]) than that in the MBCT group (2.7 [2.4]). No significant difference was observed in the dose of all drug categories between the treatment arms at any point in the study (**Table 2**). No serious adverse events occurred during the study period. 3.3% of the EQ-5D data were missing.

**Table 2:** Treatment engagement by study group.

	MBCT (n = 20)	TAU (n = 20)	p value*
No. of MBCT sessions attended, mean (SD)	6.8 ( 2.2 )		
Completion rate of the full course of MBCT sessions, n (%)	12 ( 60 )		
No. of psychiatrist visits, mean (SD)			
Between 4 wk before baseline and baseline	1.7(1.3)	1.9(1.1)	0.606
Between baseline and 4 wk	1.2(1.2)	1.9(1.2)	0.092
Between 4 and 8 wk	1.5(1.3)	1.6(1.0)	0.680
Between baseline and 8 wk	2.7(2.4)	3.5(2.0)	0.262
Medication			
Antidepressants dose (DDD) at each time point, mean (SD), wk			
0 (baseline)	0.93(0.77)	1.39(0.97)	0.107
4	0.90(0.78)	1.29(0.90)	0.157
8	0.95(0.80)	1.14(0.89)	0.487
Anxiolytics/hypnotics dose (DDD) at each time point, mean (SD), wk			
0 (baseline)	0.94(0.86)	1.34(1.54)	0.322
4	0.84(0.87)	1.14(1.41)	0.413
8	0.91(0.89)	1.23(1.48)	0.408
Antipsychotics dose (DDD) at each time point, mean (SD), wk			
0 (baseline)	0.06(0.15)	0.06(0.15)	0.972
4	0.05(0.14)	0.05(0.12)	0.936
8	0.06(0.15)	0.06(0.14)	0.943
Mood stabilizer dose (DDD) at each time point, mean (SD), wk			
0 (baseline)	0.22(0.47)	0.18(0.31)	0.741
4	0.23(0.50)	0.17(0.31)	0.681
8	0.18(0.39)	0.16(0.30)	0.843

*The p values reported from t-test.

MBCT, mindfulness-based cognitive therapy; DDD, defined daily dose; TAU, treatment as usual; SD, standard deviation.

This is the intention-to-treat-analysis.

### 3.3 Cost and effectiveness consequences

**Table 3** shows the mean costs and clinical outcomes and the difference between the groups (n=38 with complete data). The analysis was conducted with a mixed-effects model repeated-measures approach. The model included intervention group, week, group-by-week interaction, age, and sex as fixed effects.

**Table 3:** Cost and effectiveness consequence analysis.

All patients (n = 38)			
Cost	0-8 weeks		Difference over an entire period (0-8weeks) (95% CI)
	MBCT (n=19)	TAU (n=19)	
Visit cost			
JPY	13,547(12,107)	17,716(10,213)	-4,168 ( -11,538 to 3,201)
USD	129(115)	169(97)	-40 ( -110 to 31)
Drug cost			
JPY	5,976(12,107)	7,239(10,213)	-1,264 ( -4,699 to 2,172)
USD	57(115)	69(97)	-12 ( -45 to 21)
MBCT cost			
JPY	19,326(4,612)	NA	NA
USD	149(36)	NA	NA
Total			
JPY	38,850(12,107)	24,955(11,410)	13,894 ( 5,926 to 21,863)
USD	371(122)	238(109)	133 ( 57 to 209)

Effectiveness*	Week 8		Difference over an entire period (0-8weeks) (95% CI)
	MBCT (n=19)	TAU (n=19)	
Change of STAI-S	-10.368(2.317)	-0.316(2.317)	-10.053(-16.475 to -3.630 )
Change of STAI-T	-11.684(2.250)	0.211(2.250)	-11.895(-18.132 to -5.658 )
Change of EQ-5D	0.050(0.036)	-0.011(0.036)	0.061(-0.038 to 0.161 )

MBCT, mindfulness-based cognitive therapy; TAU, treatment as usual; CI, confidence interval,

JPY, Japanese yen, USD: United States dollars; STAI, state-trait anxiety inventory; EQ-5D, EuroQoL five-dimensional questionnaire

Values are expressed as the means and standard deviations unless stated otherwise.

*Results of a mixed-effects model for repeated measures.

Although the psychiatrist visit count and medication costs were lower in the augmented MBCT group than in the TAU group, the augmented MBCT group spent JPY 13,894 more than the TAU group because of the additional MBCT cost, which showed statistical significance.

Regarding the clinical outcomes, the scores of STAI-S, and STAI-T in the MBCT significantly reduced (mean [SD])(STAI-S: -10.368 [2.317], STAI-T: -11.684 [2.250]), while those in the TAU was almost constant (STAI-S: -0.316 [2.317], STAI-T: 0.211 [2.250]). Therefore, the difference between the groups at week 8 was significant (STAI-S: −10.053 [3.277] p=0.003, STAI-T: −11.895 [3.182] p<0.001). In the health utility domain of the EQ-5D questionnaire, the score of the augmented MBCT group was 0.061 higher than that of the TAU group; however, the difference was not significant (p=0.23).

### 3.4 Cost effectiveness

#### 3.4.1 ICER per STAI

**Figure 2:**
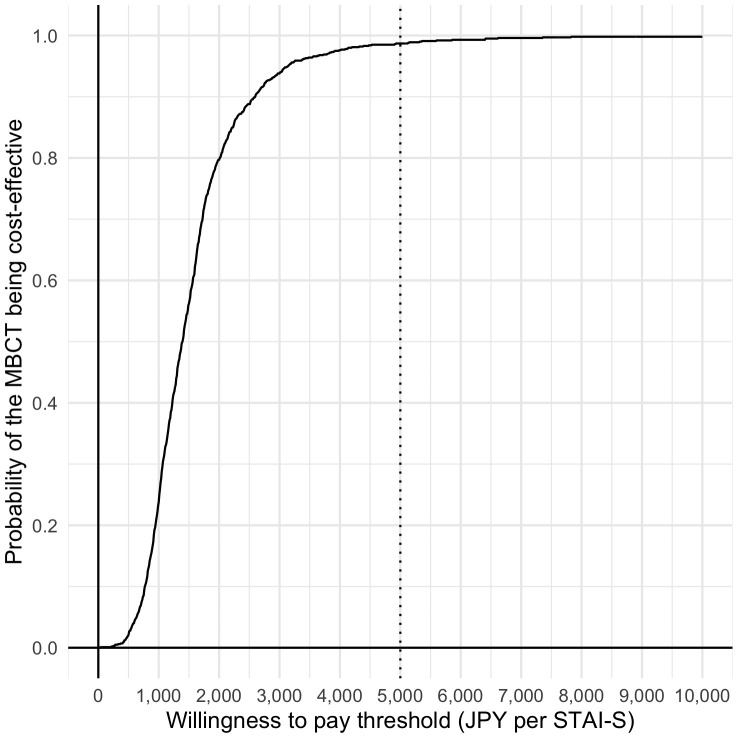
Cost-effectiveness acceptability curves (STAI-S).

**Figure 3:**
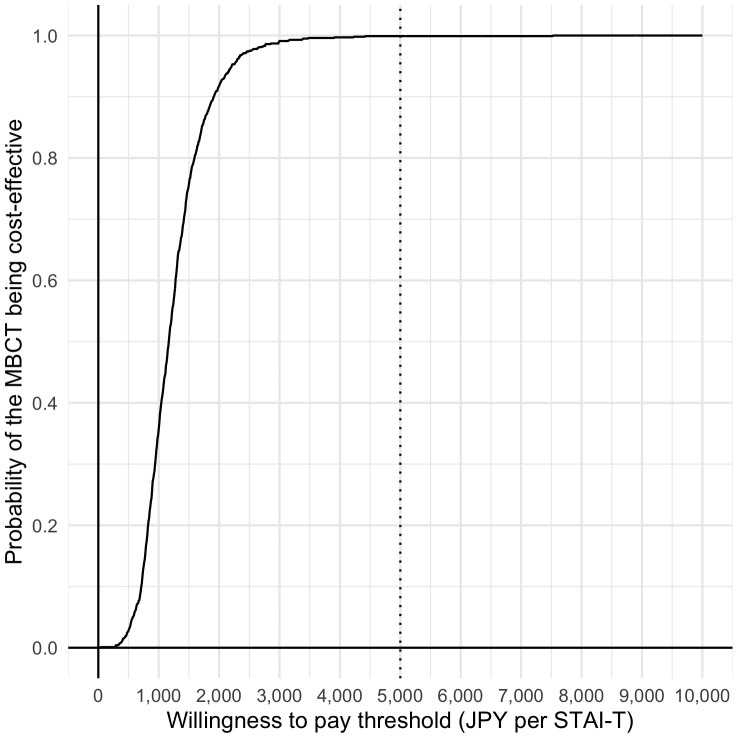
Cost-effectiveness acceptability curves (STAI-T).

A total of 38 participants, with complete data, were included in the base case cost-effectiveness analysis. The MBCT was JPY 13,885 more costly than the cost of TAU and the respective decrements in STAI-state and STAI-trait were 10.13 and 12.00. Then, the ICERs were JPY 1,371 per 1-point decrement in STAI-S and JPY 1,157 per 1-point decrement in STAI-T score, respectively (**Table 4**). The relevant acceptability curves are shown in **Figures 2**, **3**. Although no established WTP threshold of ICER for STAI available in Japan, the probabilities that MBCT is cost-effective at a WTP of JPY 5,000 per 1-point STAI-S, -T improvement were 98.7% and 99.9% respectively.

**Table 4:** Cost-effectiveness analysis of the base case.

Effect	Mean differences (95% CI) and ICERs
	(n =38: MBCT 19 vs TAU 19)
Incremental costs(JPY)	13,885(6,376 to 21,969)
(USD)	132(61 to 210)
Incremental STAI-S decrement	10.13 (4.2 to 16.05)
ICER (JPY per STAI-S)	1,371
(USD per STAI-S)	13
Incremental STAI-T decrement	12.00(6.15 to 17.80)
ICER (JPY per STAI-T)	1,157
(USD per STAI-T)	11
Incremental QALY gain	0.009(-0.006 to 0.025)
ICER (JPY per QALY)	1,566,357
(USD per QALY)	14,940

ICER, incremental cost-effectiveness ratio; QALY, quality-adjusted life years,

CI, confidence interval; MBCT, mindfulness-based cognitive therapy; TAU, treatment as usual,

STAI-S, state-trait anxiety inventory (state); STAI-T, state-trait anxiety inventory (trait),

JPY, Japanese yen; USD, United States dollars.

These values were estimated based on the data of the complete samples.

QALY was estimated based on EQ5D.

#### 3.4.2 ICER per QALY gained

**Figure 4:**
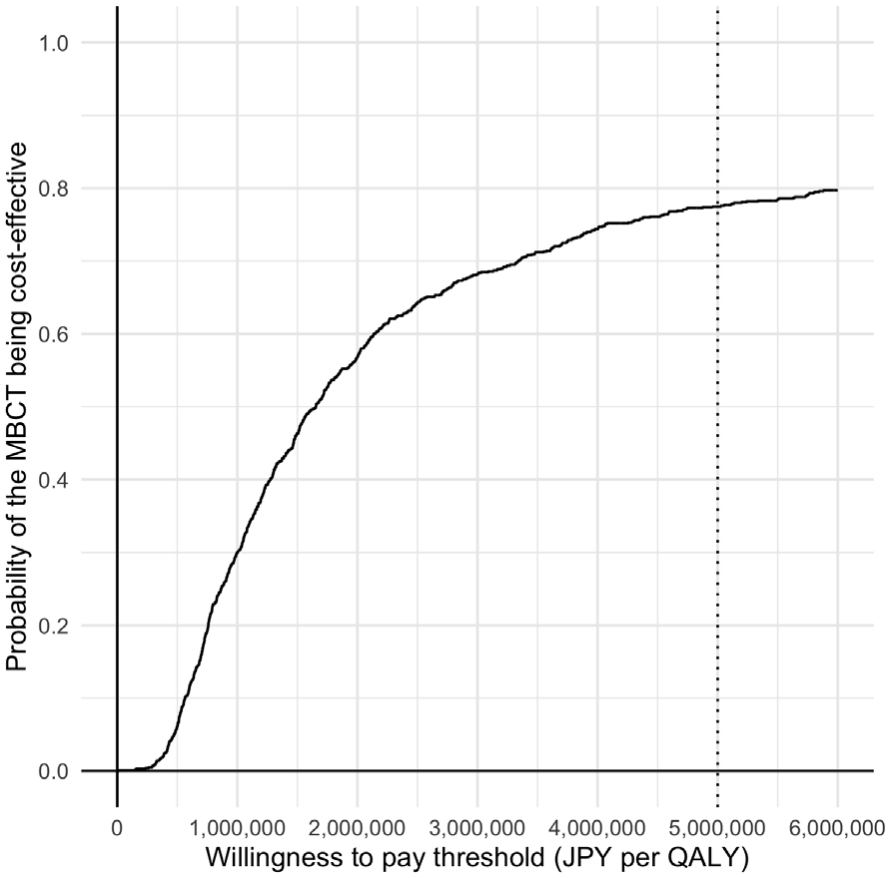
Cost-effectiveness acceptability curves (QALY).

Same as the analysis for ICER per STAI, a total of 38 participants, with complete data, were included in the analysis (**Table 4**). The incremental cost remained the same as in the previous analysis (JPY 13,885) and was associated with a QALY increase of 0.009. Therefore, the ICER was JPY 1,566,357 (USD 14,940) below the threshold adopted in Japan (i.e., JPY 5,000,000 (USD 47,692) per QALY gained) (43). The acceptability curve (**Figure 4**) demonstrated an 77.5% probability that MBCT is cost-effective at a WTP threshold of JPY 5,000,000 (USD 47,692) per QALY.

### 3.5 Sensitivity analyses

#### 3.5.1 ICER per STAI

In the first scenario with the MBCT cost of JPY 32,529/session (n=38: augmented MBCT 19 vs. TAU 19), The MBCT was JPY 20,618 more costly than the cost of TAU and the respective decrement in STAI-state and STAI-trait were 10.05 and 11.90. Then, the ICERs were JPY 2,052 (USD 20) per STAI-S and JPY 1,733 (USD 17) per STAI-T, respectively (**Supplementary Table 2**). The relevant acceptability curves are shown in **Supplementary Figures 1**, **2**. The probabilities that MBCT is cost-effective at a WTP of JPY 5,000 per 1-point STAI-S, -T decrement were 96.5% and 99.8%.

In the second scenario, analysis was conducted on all participants, including those with missing values (n=40: augmented MBCT 20 vs. TAU 20). LOCF was performed for the imputation of the missing STAI scores (no imputation was performed for the cost). The MBCT was JPY 13,780 more costly than the cost of TAU and the respective incremental decrement in STAI-state and STAI-trait were 10.22 and 11.83. Then, the ICERs were JPY 1,348 (USD 13) per STAI-S and JPY 1,165 (USD 11) per STAI-T, respectively (**Supplementary Table 3**). The relevant acceptability curves are shown in **Supplementary Figures 3**, **4**. The probabilities that MBCT is cost-effective at a WTP of JPY 5,000 per STAI decrement were 98.7% and 99.9% respectively.

The third sensitivity analysis with an MBCT unit cost of JPY 32,529/session, including the samples with missing values (n=40: augmented MBCT 20 vs. TAU 20), revealed that the ICER per STAI-S, and per STAI-T were JPY 2,106 (USD 20), and JPY 1,751 (USD 17) respectively (**Supplementary Table 4**). The augmented MBCT had an 94.6% and 99.5% probability of being cost-effective at a WTP of JPY 5,000 per 1-point STAI improvement (**Supplementary Figures 5**, **6**).

#### 3.5.2 ICER per QALY

In the first scenario with the MBCT cost of JPY 32,529/session (n=38: augmented MBCT 19 vs. TAU 19), the ICER became JPY 2,364,440 (USD 22,553) per QALY (**Supplementary Table 2**), which was below the WTP threshold in Japan (JPY 5,000,000 (USD 47,692) per QALY). The acceptability curve indicated that the probability of the augmented MBCT being cost-effective was 72.5% at a WTP threshold of JPY 5,000,000 (USD 47,692) per QALY (**Supplementary Figure 7**).

In the second scenario, analysis was conducted on all participants, including those with missing values (n=40: augmented MBCT 20 vs. TAU 20). LOCF was performed for the imputation of the missing EQ-5D scores (no imputation was performed for the cost). The incremental QALY remained 0.009, while the incremental cost became JPY 13,780, leading to a slightly preferable ICER (JPY 1,487,395 (USD 14,187) per QALY) (**Supplementary Table 3**). The augmented MBCT was 79.2% cost-effective at a WTP threshold of JPY 5,000,000 (USD 47,692) per QALY (**Supplementary Figure 8**).

The third sensitivity analysis with an MBCT unit cost of JPY 32,529/session, including the samples with missing values (n=40: augmented MBCT 20 vs. TAU 20), revealed that the ICER was JPY 2,409,698 (USD 22,985) per QALY (**Supplementary Table 4**). The augmented MBCT had an 71.3% probability of being cost-effective at a WTP threshold of JPY 5,000,000 (USD 47,692) per QALY (**Supplementary Figure 9**).

The fourth sensitivity analysis using the incremental area of QALYs from the baseline method, with complete samples (n=38: augmented MBCT 19 vs. TAU 19), revealed that the ICER was JPY 2,408,526 (USD 22,973) per QALY (**Supplementary Table 5**). The augmented MBCT had an 71.2% probability of being cost-effective at a WTP threshold of JPY 5,000,000 (USD 47,692) per QALY (**Supplementary Figure 10**).

## 4 Discussion

### 4.1 Overall findings

This study is the first to evaluate the cost-effectiveness of augmented MBCT for anxiety disorders. This study is unique because it 1) was conducted in a secondary mental healthcare setting, where the treatment duration of the participants was quite long, and 2) compared the cost-effectiveness of augmented MBCT with that of TAU alone (i.e., pharmacotherapy: the most prevalent treatment strategy).

Regarding the ICER per STAI, there is no established consensus on a WTP threshold. To the best of our knowledge, no studies have reported the ICER per STAI improvement for anxiety disorders. However, a study evaluating the cost-effectiveness of augmented CBT for treating moderate to severe depression in Japan (44) reported ICERs per 1-point decrement in the GRID-HDRS17, BDI-II, and QIDS scales ranging from JPY 14,262 to 36,166. Although the score ranges of these scales differ, the ICER per STAI (approximately JPY 1,400 per STAI) was notably lower. In the intervention group of this study, the mean STAI scores improved by approximately 10 points for STAI-S and 12 points for STAI-T, approaching the normal range. These findings suggest that such near-remission improvements can be achieved at a cost of approximately JPY14,000, thereby demonstrating the cost-effectiveness of MBCT.

Although the QALY improvement did not reach the statistically significance, the ICER per QALY showed favorable results. The base analysis indicated that MBCT is cost-effective because the ICER [i.e., JPY 1,566,357 (USD 14,940)] is well below the WTP threshold (JPY 5,000,000), and the probability of MBCT being cost-effective is 0.775. Because the results remained the same even after conducting a series of sensitivity analyses (i.e., ICERs were between JPY 1,487,395 (USD 14,187) and JPY 2,409,698 (USD 22,985); and the probabilities of MBCT being cost-effective were between 0.712 and 0.792, the finding that the augmented MBCT is cost-effective is quite robust.

In this study, as previously noted, while both STAI-S and STAI-T showed significant improvement, the incremental QALYs did not reach statistical significance. This may be attributed to the use of the EQ-5D-3L, as previous research suggests that its responsiveness (i.e. the ability to accurately capture change of symptoms overtime) to anxiety disorders is less pronounced compared to depression (45). However, since responsiveness has improved in the EQ-5D-5L (46), the results might have differed had the EQ-5D-5L been used.

### 4.2 Comparison with other studies

Unfortunately, previous systematic reviews (26, 27) were unable to find studies that investigated the cost-effectiveness of MBIs for anxiety disorders. The only relevant study was that conducted by Saha et al. (28), who assessed the cost-effectiveness of MBCT and compared it with that of CBT in the clinical population, including those with anxiety. This study reported that mindfulness group therapy significantly reduced healthcare costs, despite showing no significant difference in effectiveness compared to the control group. However, this trial involved a patient population with various diagnoses (e.g., depression, anxiety, stress, and adjustment disorders), and 76% of the control group participants received individual CBT. Therefore, the results of the present study were difficult to directly compare with those of similar studies.

On the other hand, expanding the scope to other conditions enabled the conduct of further discussions. Shawyer et al. (47) evaluated the cost-effectiveness of the augmented MBCT for preventing new episodes in patients with recurrent major depression. This study was conducted alongside an RCT comparing augmented MBCT (MBCT plus active monitoring) with active monitoring alone for 2 years. Findings showed that MBCT resulted in better outcomes and was less costly (the ICER was AUD 83,744, below the threshold in Australia [AUD 100,000]), highlighting the augmented dominance of MBCT. Similar results were observed in patients with other conditions such as multiple sclerosis (48). In the trial, MBCT was not superior to the waitlist in terms of the QALY gained. However, because healthcare costs were substantially reduced, the probability of MBCT being cost-effective was quite high (87.4% with a WTP threshold of GBP 20,000). These and our findings imply that MBCT is likely to be cost-effective when provided in addition to usual care, irrespective of clinical conditions.

### 4.3 Clinical and policy implications

Augmented MBCT is effective and cost-effective compared with TAU for anxiety disorders. However, considering some constraints of the study, such as small sample size, and short study duration combined with the chronic nature of anxiety disorders and concerns raised about the sensitivity of the measurement instruments over such a period, further research over longer period with larger and more representative samples is required to establish more definitive conclusions.

Another policy implication is that a training system for MBCT therapists should be established. The growth of well-trained psychotherapists requires time, cost, and a well-designed system. With the adoption of the “improving access to psychological treatment” program in the UK (49), a well-organized and systematic training program for therapists is essential. In Japan, the Japanese Ministry of Health, Labour and Welfare has provided opportunities for CBT trainees to attend workshops and receive supervision since 2011 (50). A similar system is required to disseminate MBCT to patients.

In addition, a precise estimate of the unit cost for human resources required to provide group psychotherapy is needed. Under the public health insurance system in Japan, group psychotherapy is reimbursed at a uniform rate regardless of the therapist’s degree or level of proficiency. Therefore, from the perspective of insurance-based analysis, these differences in conditions do not affect the results. However, to provide a more accurate estimate, such data is essential.

### 4.4 Strengths and limitations

We found the strength of the study in using multiple effectiveness measures (i.e.STAI, and EQ-5D); having an excellent retention rate (95%); contributing to a rare yet important literature area by using best practices to calculate costs alongside effectiveness in a randomized controlled trial of psychotherapy. Furthermore, by clarifying the cost-effectiveness of MBCT as a group-based psychotherapy, the study offers important insights into expanding access to psychotherapy for patients in need, particularly in settings with limited human resources. However, we also acknowledge some limitations. The first was related to the representativeness of typical patient population. It was constrained due to the study participants being selected only from university hospital patients with a dominance of males over females (70% in the intervention arm and 55% in the control group). Second, Using the wait-list group as a control group may have potentially biased the results in a way that makes them appear larger. Third, the observational period was relatively short (i.e., 8 weeks). One study reported that the MBCT’s effect observed within a short term disappeared in the long term (51); therefore, the assessment of the long-term effect is important. Fourth, the EQ-5D-3L was used in the trial because the EQ-5D-5L was unavailable at the beginning of the study. As the EQ-5D-5L improves the ceiling effect, the difference might have been observed if we had been able to use the EQ-5D-5L instead. Additionally, using other scales such as the SF-6D or HUI might have produced different results. Fifth, we did not conduct an analysis from a societal perspective. This is solely because work productivity was not assessed during the trial. However, economic evaluation from a societal perspective would yield different interpretations than those from a public healthcare insurance perspective. Sixth, inability to conceal the participants or the MBCT therapists’ allocation status might have biased self- reported outcomes, with participants in the MBCT group possibly overestimating benefits and those in the control group underestimating them due to perceived inferiority. Seventh, we collected the data on healthcare service use via self-report. Then, we could not reduce the effects of recall bias. Therefore, we should be aware that these limitations would impact the wider applicability of the findings. Future research should consider these issues to improve the quality of economic evaluations relevant to MBCT for anxiety disorders.

## 5 Conclusion

In conclusion, augmented MBCT for anxiety disorders is cost-effective considering WTP threshold for ICER per QALY in Japan (JPY 5,000,000) at 8-week compared with TAU (pharmacotherapy) post-treatment from a public healthcare insurance perspective. Therefore, future studies should conduct a long-term observation, use a larger sample size, and perform an analysis from a societal perspective.

## Data Availability

The disclosure of the raw data supporting the conclusions of this article requires the ethical committee’s permission. Requests to access the datasets should be directed to MS, mitsusado@keio.jp.

## Supplementary material

Supplementary Figure 1 Cost-effectiveness acceptability curves (STAI-S) with sensitivity analysis 1.

Supplementary Figure 2 Cost-effectiveness acceptability curves (STAI-T) with sensitivity analysis 1.

Supplementary Figure 3 Cost-effectiveness acceptability curves (STAI-S) with sensitivity analysis 2.

Supplementary Figure 4 Cost-effectiveness acceptability curves (STAI-T) with sensitivity analysis 2.

Supplementary Figure 5 Cost-effectiveness acceptability curves (STAI-S) with sensitivity analysis 3.

Supplementary Figure 6 Cost-effectiveness acceptability curves (STAI-T) with sensitivity analysis 3.

Supplementary Figure 7 Cost-effectiveness acceptability curves (QALY) with sensitivity analysis 1.

Supplementary Figure 8 Cost-effectiveness acceptability curves (QALY) with sensitivity analysis 2.

Supplementary Figure 9 Cost-effectiveness acceptability curves (QALY) with sensitivity analysis 3.

Supplementary Figure 10 Cost-effectiveness acceptability curves (QALY) with sensitivity analysis 4. JPY, Japanese yen; MBCT, mindfulness-based cognitive therapy.
