# Supplementary figures and images for: Cost-effectiveness analysis of mindfulness-based cognitive therapy in patients with anxiety disorders in secondary mental health care settings alongside a randomized controlled trial
Source: Front Psychiatry. 2024 Oct 25;15:1391786. doi: 10.3389/fpsyt.2024.1391786 (PMC11544232; doi:10.3389/fpsyt.2024.1391786)

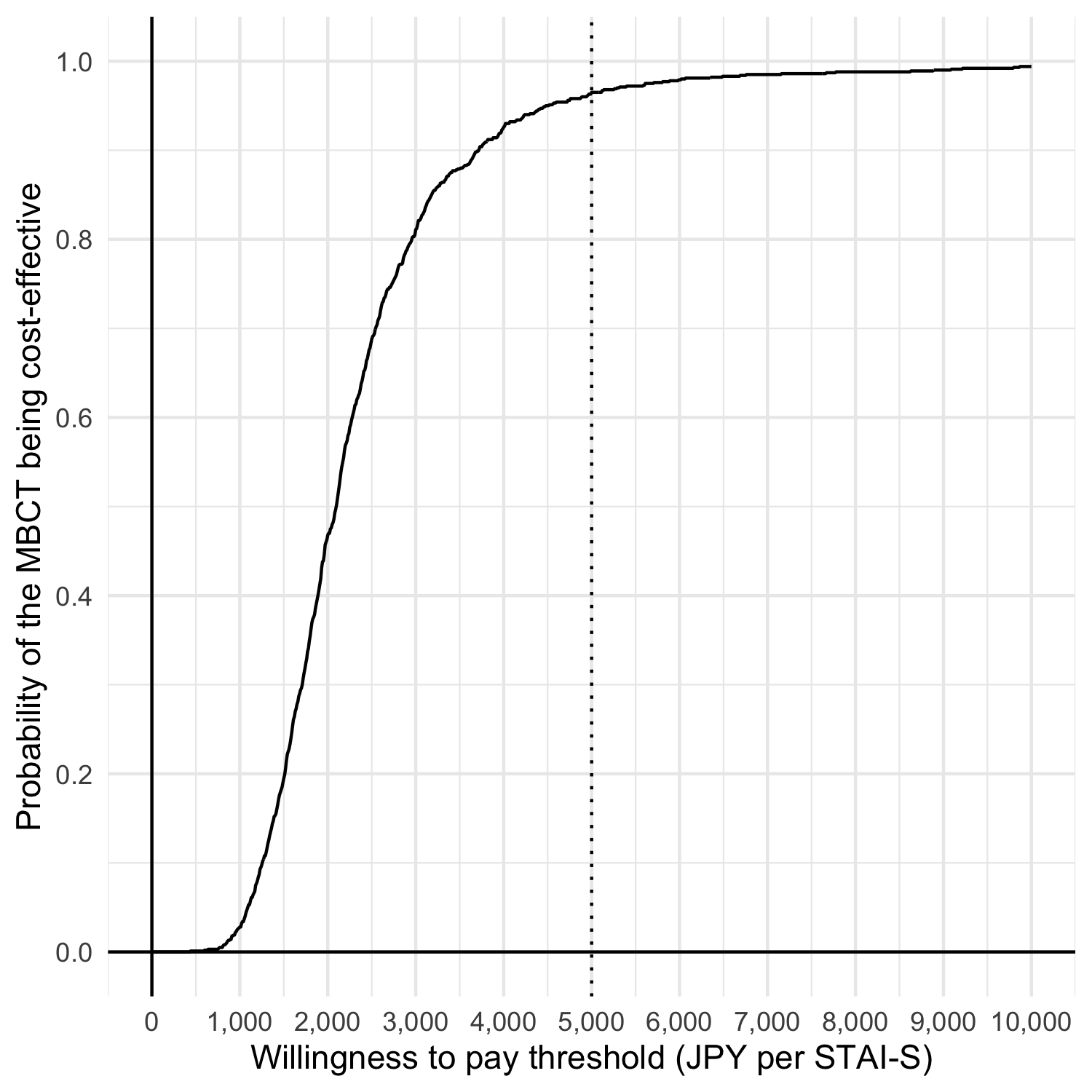

Supplement: Supplementary Figure 1 — Cost-effectiveness acceptability curves (STAI-S) with sensitivity analysis 1. [file DataSheet1.zip › Supplementary Figure 1.TIFF]

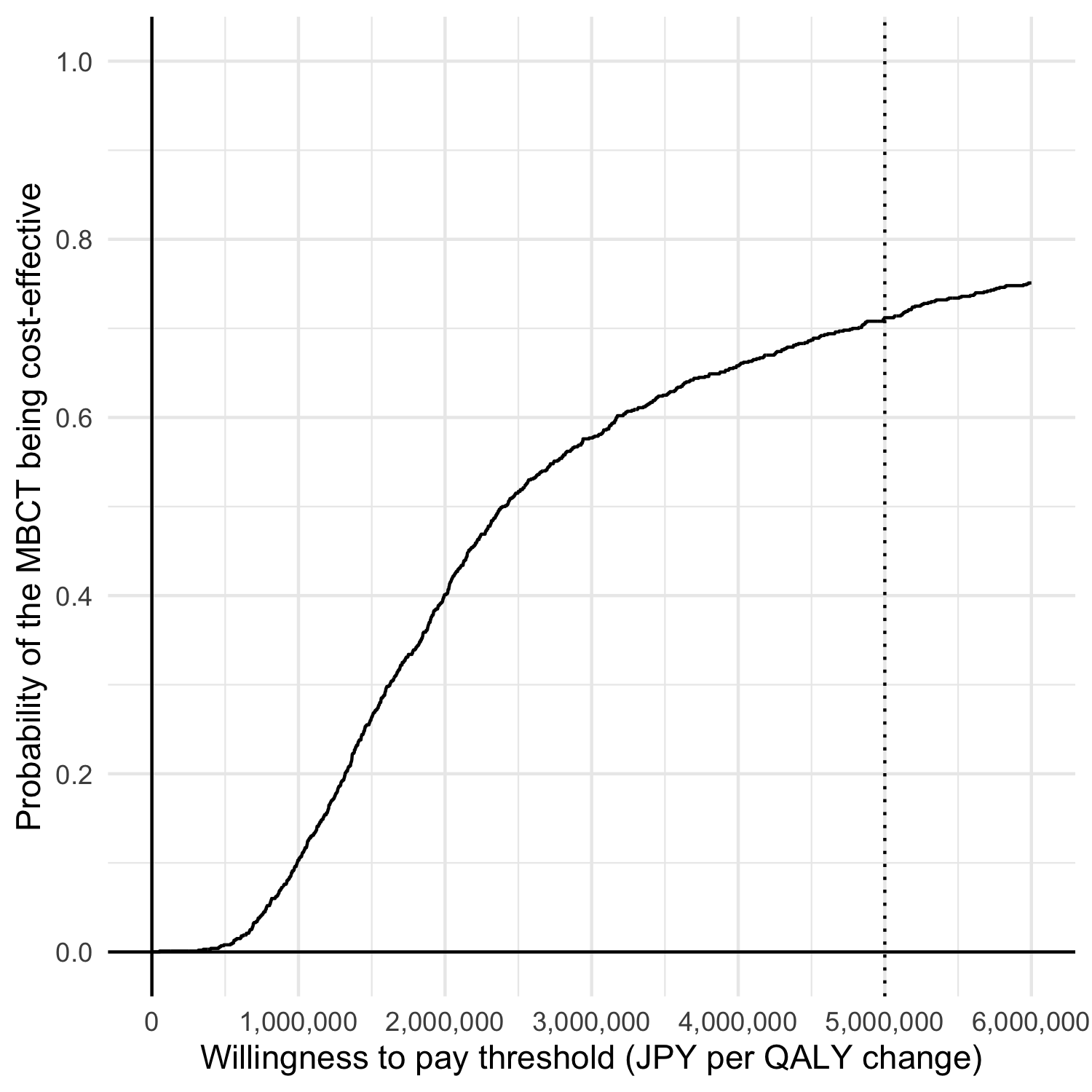

Supplement: Supplementary Figure 1 — Cost-effectiveness acceptability curves (STAI-S) with sensitivity analysis 1. [file DataSheet1.zip › Supplementary Figure 10.TIFF]

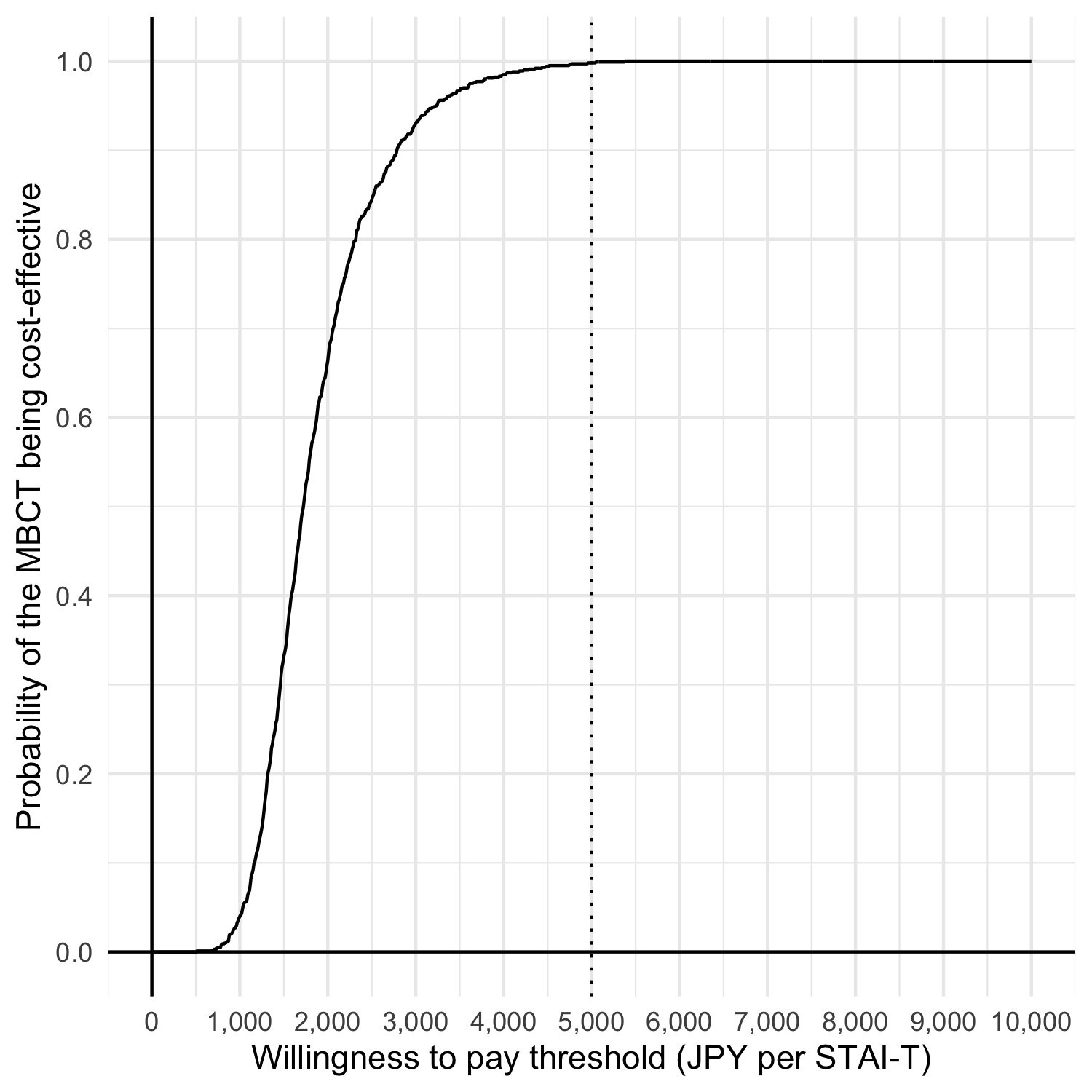

Supplement: Supplementary Figure 1 — Cost-effectiveness acceptability curves (STAI-S) with sensitivity analysis 1. [file DataSheet1.zip › Supplementary Figure 2.TIFF]

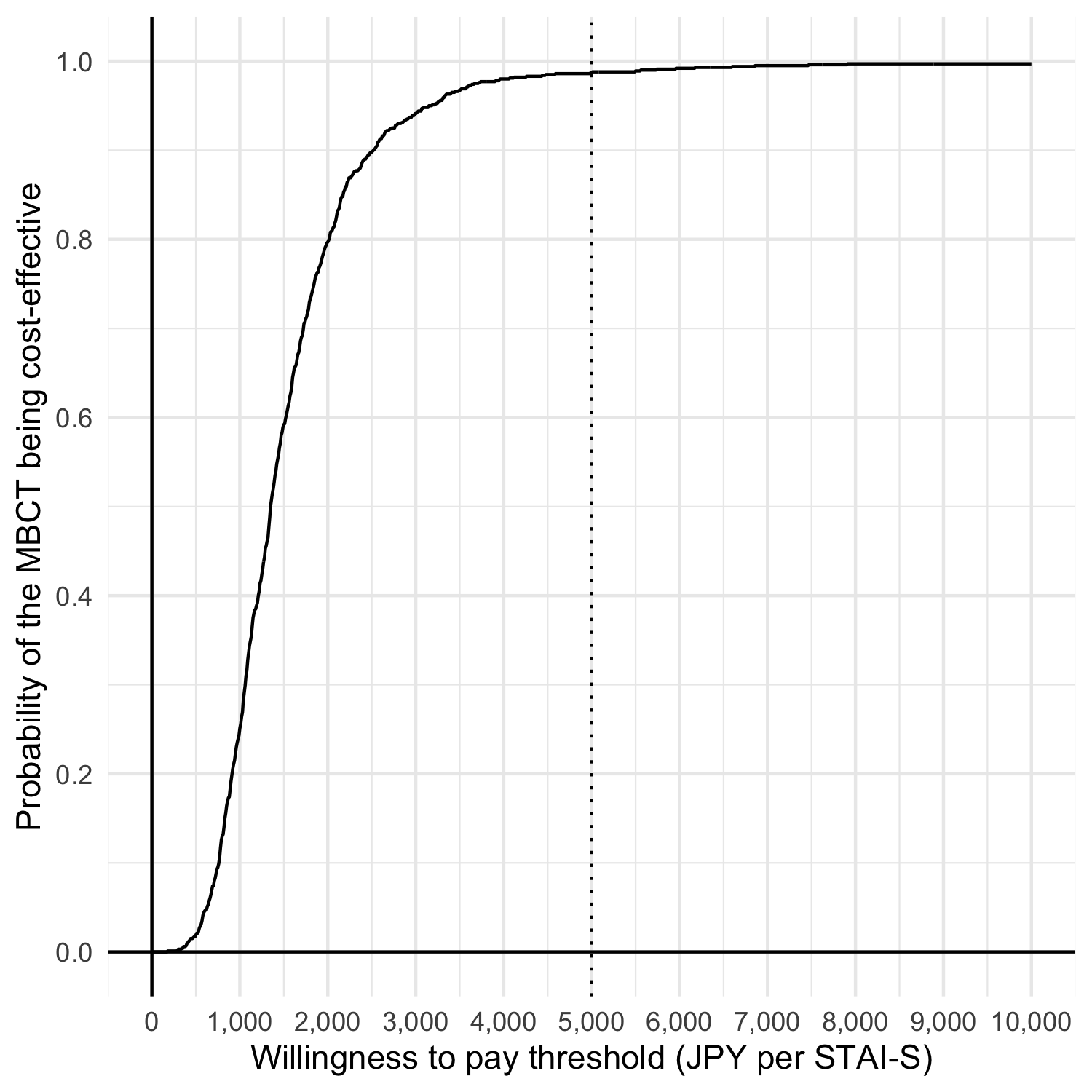

Supplement: Supplementary Figure 1 — Cost-effectiveness acceptability curves (STAI-S) with sensitivity analysis 1. [file DataSheet1.zip › Supplementary Figure 3.TIFF]

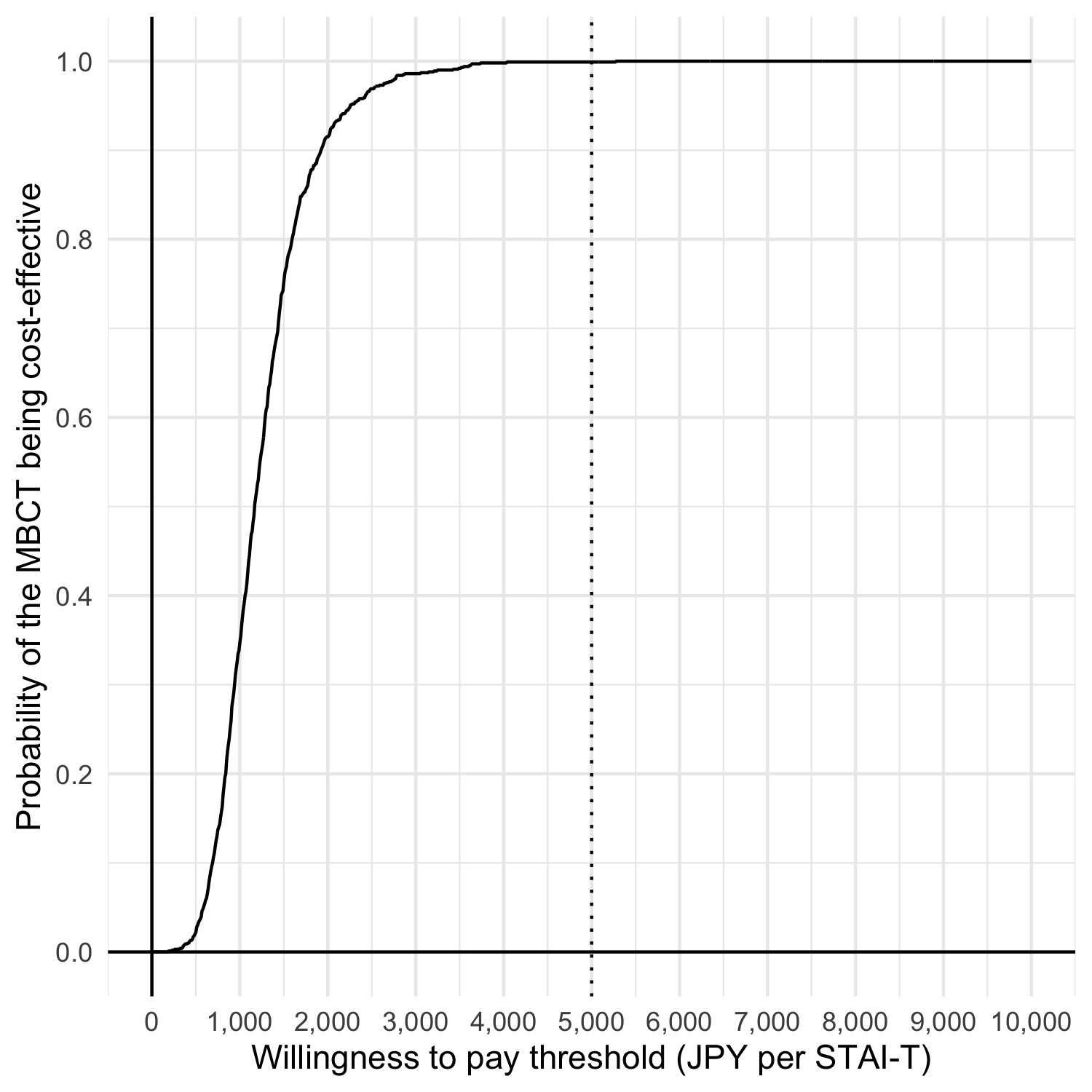

Supplement: Supplementary Figure 1 — Cost-effectiveness acceptability curves (STAI-S) with sensitivity analysis 1. [file DataSheet1.zip › Supplementary Figure 4.TIFF]

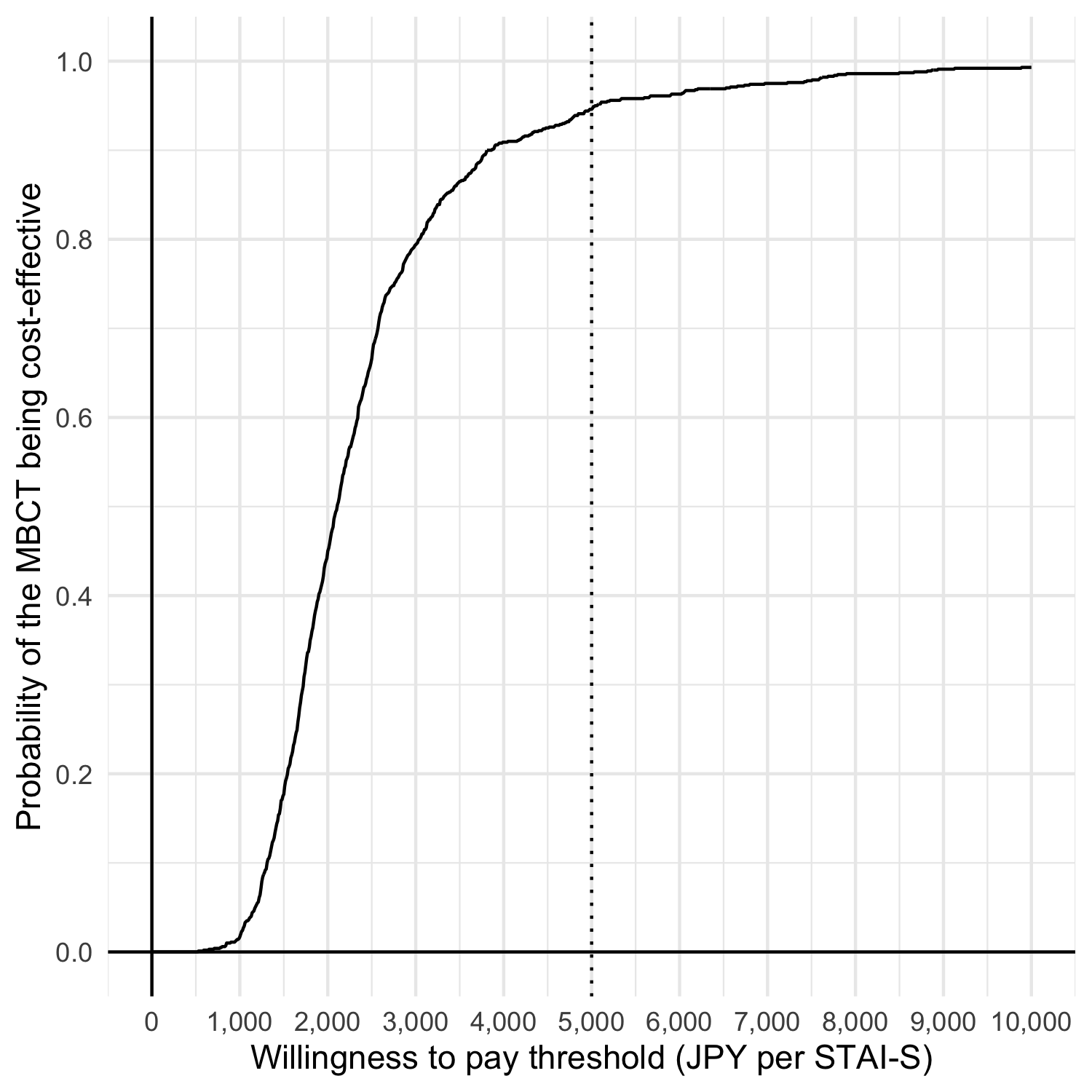

Supplement: Supplementary Figure 1 — Cost-effectiveness acceptability curves (STAI-S) with sensitivity analysis 1. [file DataSheet1.zip › Supplementary Figure 5.TIFF]

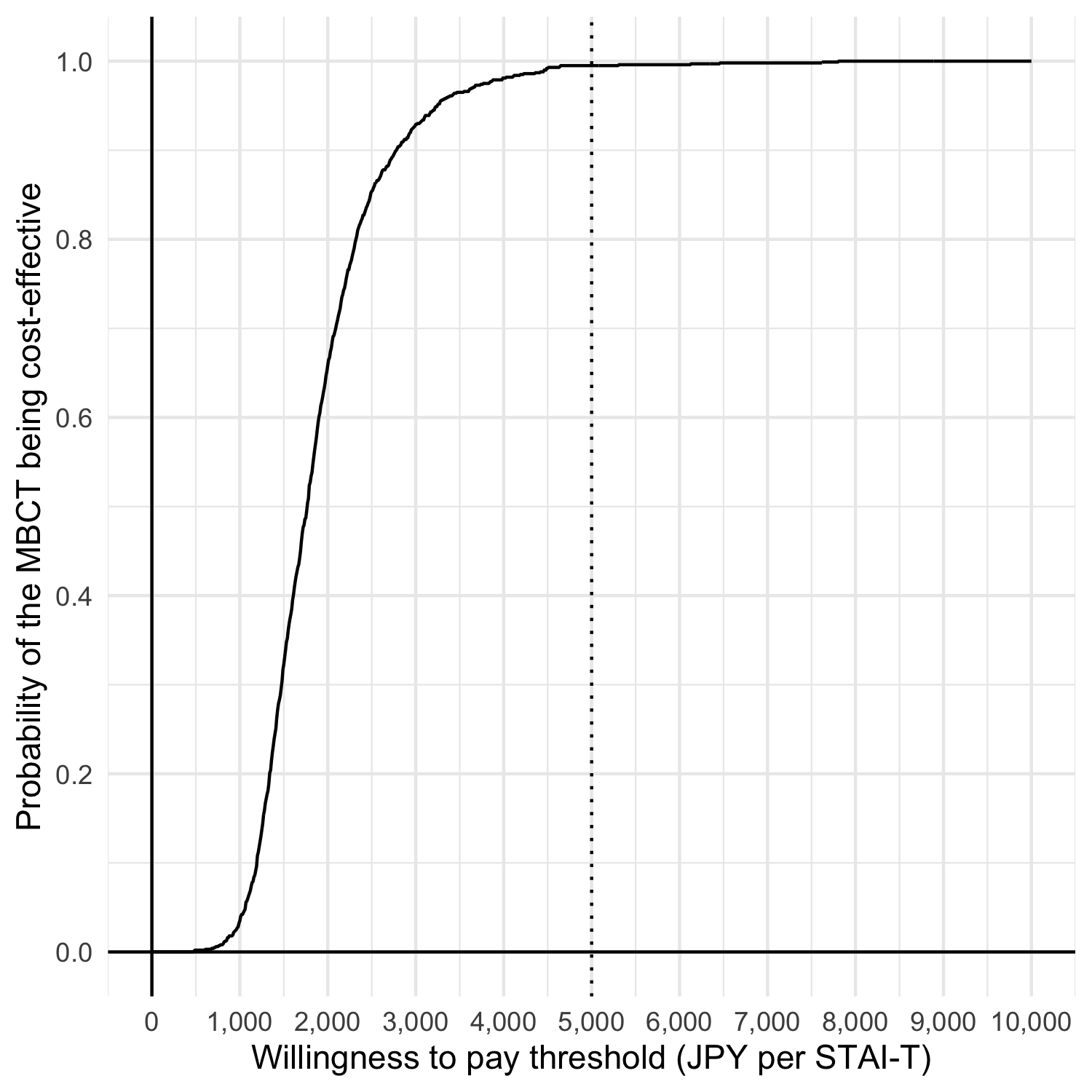

Supplement: Supplementary Figure 1 — Cost-effectiveness acceptability curves (STAI-S) with sensitivity analysis 1. [file DataSheet1.zip › Supplementary Figure 6.TIFF]

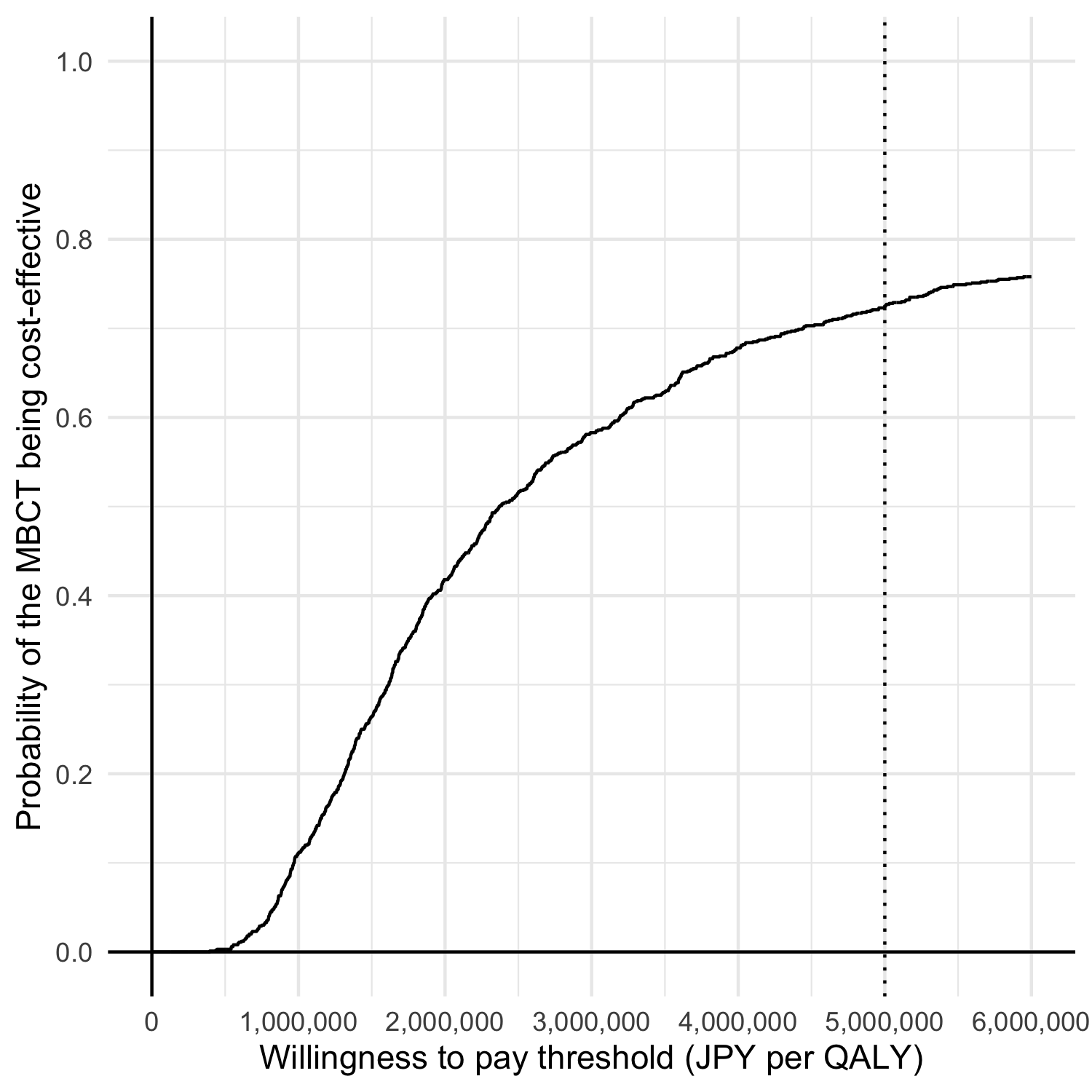

Supplement: Supplementary Figure 1 — Cost-effectiveness acceptability curves (STAI-S) with sensitivity analysis 1. [file DataSheet1.zip › Supplementary Figure 7.TIFF]

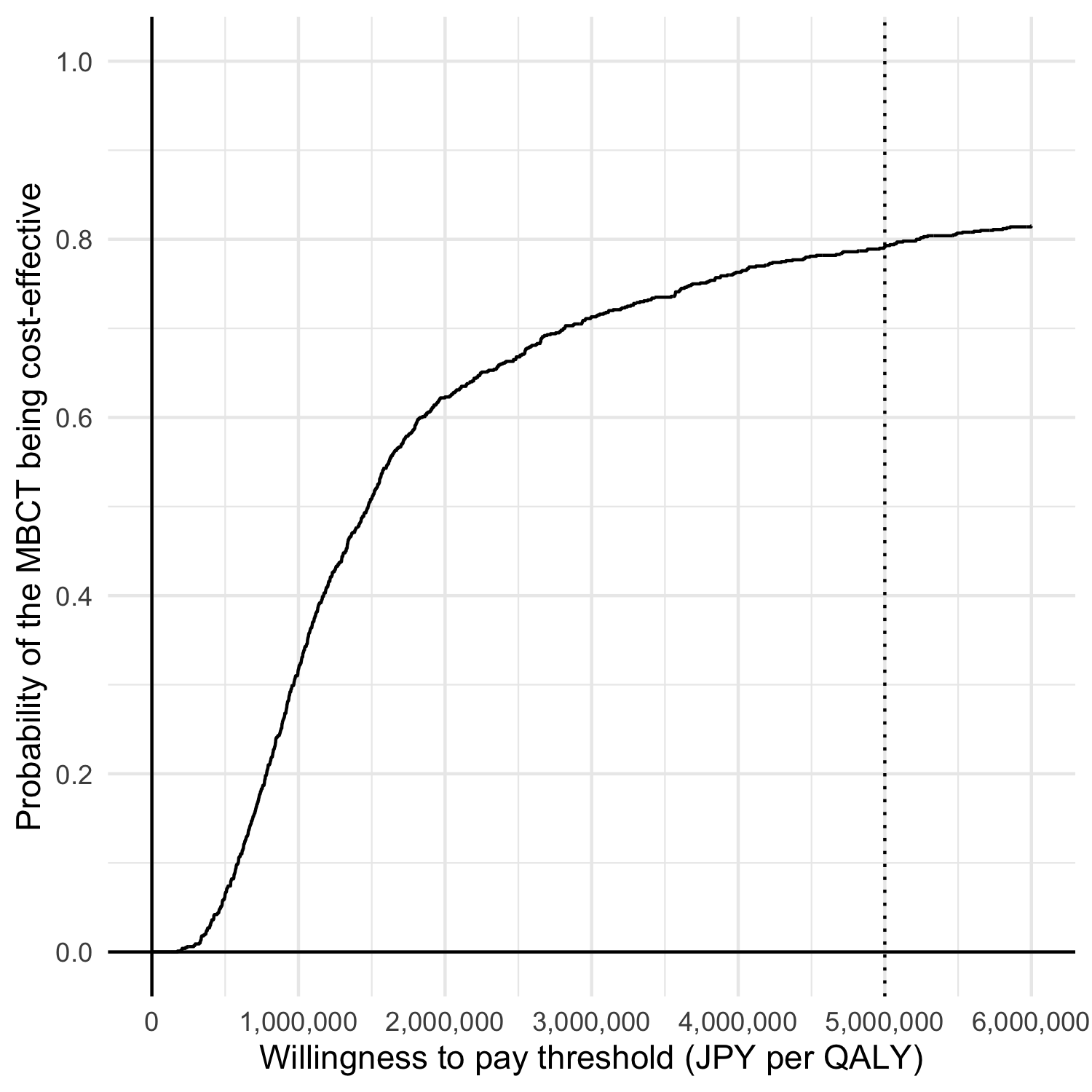

Supplement: Supplementary Figure 1 — Cost-effectiveness acceptability curves (STAI-S) with sensitivity analysis 1. [file DataSheet1.zip › Supplementary Figure 8.TIFF]

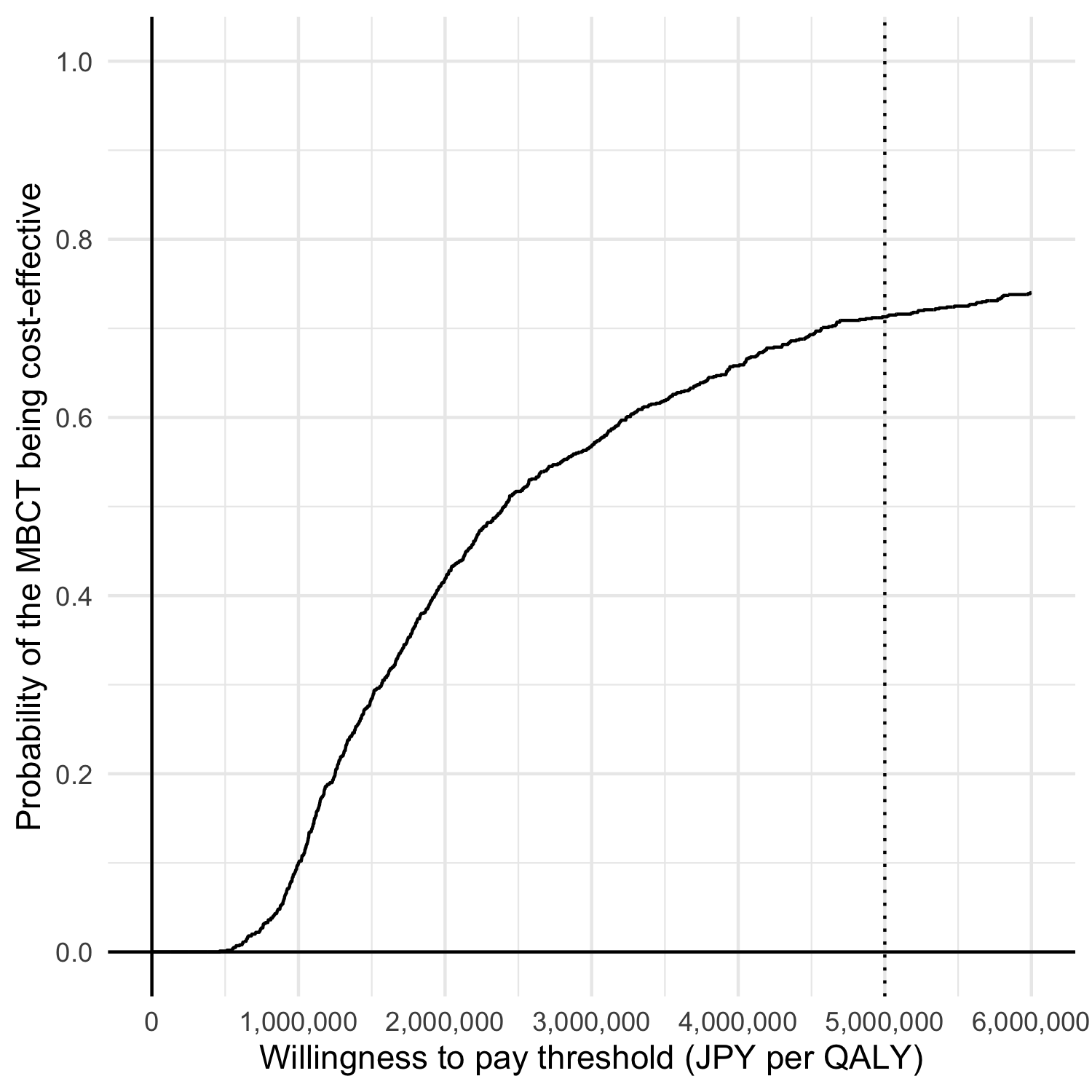

Supplement: Supplementary Figure 1 — Cost-effectiveness acceptability curves (STAI-S) with sensitivity analysis 1. [file DataSheet1.zip › Supplementary Figure 9.TIFF]
